# Differential fuel utilization in liver transplant recipients and its relationship with non‐alcoholic fatty liver disease

**DOI:** 10.1111/liv.15178

**Published:** 2022-02-24

**Authors:** Mohammad S. Siddiqui, Samarth Patel, Mikael Forsgren, Anh T. Bui, Steve Shen, Taseen Syed, Sherry Boyett, Shanshan Chen, Arun J. Sanyal, Susan Wolver, Danielle Kirkman, Francesco S. Celi, Chandra S. Bhati

**Affiliations:** ^1^ Division of Gastroenterology and Hepatology Virginia Commonwealth University Richmond Virginia USA; ^2^ Division of Gastroenterology and Hepatology Hunter‐Holmes McGuire VA Richmond Virginia USA; ^3^ Department of Health, Medicine and Caring Sciences Linköping University Linköping Sweden; ^4^ Department of Statistical Sciences and Operations Research Virginia Commonwealth University Richmond Virginia USA; ^5^ Division of Endocrinology, Diabetes and Metabolism Virginia Commonwealth University Richmond Virginia USA; ^6^ Department of Internal Medicine Virginia Commonwealth University Richmond Virginia USA; ^7^ Department of Kinesiology and Health Sciences Virginia Commonwealth University Richmond Virginia USA; ^8^ Division of Transplant Surgery Virginia Commonwealth University Richmond Virginia USA

**Keywords:** carbohydrates, energy expenditure, fatty acids, liver transplantation, metabolic flexibility, non‐alcoholic steatohepatitis

## Abstract

Metabolic flexibility is the ability to match biofuel availability to utilization. Reduced metabolic flexibility, or lower fatty acid (FA) oxidation in the fasted state, is associated with obesity. The present study evaluated metabolic flexibility after liver transplantation (LT).

**Methods:**

Patients receiving LT for non‐alcoholic steatohepatitis (NASH) (*n* = 35) and non‐NASH (*n* = 10) were enrolled. NASH was chosen as these patients are at the highest risk of metabolic complications. Metabolic flexibility was measured using whole‐body calorimetry and expressed as respiratory quotient (RQ), which ranges from 0.7 (pure FA oxidation) to 1.0 is (carbohydrate oxidation).

**Results:**

The two cohorts were similar except for a higher prevalence of obesity and diabetes in the NASH cohort. Post‐prandially, RQ increased in both cohorts (i.e. greater carbohydrate utilization) but peak RQ and time at peak RQ was higher in the NASH cohort. Fasting RQ in NASH was significantly higher (0.845 vs. 0.772, *p* < .001), indicative of impaired FA utilization. In subgroup analysis of the NASH cohort, body mass index but not liver fat content (MRI‐PDFF) was an independent predictor of fasting RQ. In NASH, fasting RQ inversely correlated with fat‐free muscle volume and directly with visceral adipose tissue.

**Conclusion:**

Reduced metabolic flexibility in patients transplanted for NASH cirrhosis may precede the development of non‐alcoholic fatty liver disease after LT.

## 1 INTRODUCTION

Weight gain and obesity are common after liver transplantation (LT) and are associated with increased risk of cardiovascular disease, dyslipidemia, diabetes and reduced survival after LT.^1^, ^2^, ^3^, ^4^, ^5^, ^6^ Obesity is also a known as risk factor for the development of post‐LT non‐alcoholic fatty liver disease (NAFLD), which occurs in nearly a third of patients receiving LT for non‐NAFLD cirrhosis and universally among patients receiving LT for non‐alcoholic steatohepatitis (NASH) cirrhosis.^4^, ^7^, ^8^ While chronic immunosuppression is often implicated, multiple studies have failed to show clinically significant differences in immunosuppression as the sole mediator of post‐LT weight gain, underscoring sub‐clinical derangement in certain patients that are exacerbated further with exposure to chronic immunosuppression.

Efficient energy homeostasis is central to weight maintenance and perturbations in energy homeostasis are linked to obesity and the development of NAFLD in the non‐LT population.^9^, ^10^ In normal physiology, energy metabolism is characterized by periodic shifts between glucose and fatty acid (FA) oxidation by the skeletal muscle depending on fuel availability.^11^ In the fed state, when carbohydrate supply is ample, meal‐induced insulin secretion facilitates its use as the preferred fuel source while inhibiting lipolysis and promoting the storage of excess carbohydrates as fat in adipose tissue. Conversely, in the fasted state, when dietary carbohydrate intake declines, the decrease in serum insulin levels promotes lipolysis. This results in a steady supply of FA to be used as the major fuel source during fasting.^11^, ^12^ The ability to preferentially use available biofuel for the energy‐demanding biological process is referred to as metabolic flexibility and is associated with weight maintenance.^11^ Metabolic inflexibility is the inability of skeletal muscle to preferentially utilize FA for oxidation during the fasted state and is associated with weight gain.^10^, ^13^, ^14^ Blunted FA oxidation results in the recycling of unused free FA back to adipose tissue, which is then esterified as triacylglycerol for storage, thereby, promoting adiposity.^15^

The aim of the current study was to better understand energy homeostasis and metabolic flexibility in LT recipients. As patients receiving LT for NASH cirrhosis have the greatest propensity for weight gain and development of NAFLD, we hypothesized that patients transplanted for NASH cirrhosis will have reduced metabolic flexibility compared with patients transplanted for non‐NASH indications. Furthermore, since skeletal muscle is the central organ in energy homeostasis, an inverse relationship between metabolic flexibility and skeletal muscle function will be observed.

## 2 METHODS

### 2.1 Study design and participants

The study was approved by the institutional review board (IRB) and all authors have approved the manuscript prior to submission. Adult (age ≥ 18 years) LT recipients who were at least 6 months post‐LT were invited to participate in the study. The study enrolled subjects transplanted for NASH and non‐NASH cirrhosis. The diagnosis of NASH as aetiology of cirrhosis requiring LT was established if patients had (1) a prior biopsy showing NASH with progression to cirrhosis, (2) evidence of steatohepatitis or steatosis on explant or (3) prior history of metabolic co‐morbid conditions even if explant did not show NASH/NAFLD (i.e. burnt out NASH) after a negative serological evaluation and less than moderate alcohol consumption.^16^ Patients transplanted for non‐NASH cirrhosis who developed post‐LT NAFLD as evidenced by controlled attenuation parameter on vibration‐controlled transient elastography greater than 270 dB/m were excluded in this proof of concept study to avoid the potential confounders from post‐LT de novo NAFLD.^17^ Additional exclusion criteria included multi‐organ transplants, renal failure requiring haemodialysis, prednisone use, gastroparesis, non‐dermatological malignancy and poorly controlled diabetes defined by HbA1c > 8.5%. Patients with acute/chronic rejection, vascular and biliary complications within 6 months of screening were also excluded.

### 2.2 Study procedures

After enrolment, all patients were admitted to the Clinical Research Service Unit (CRSU). Study participants were instructed to abstain from strenuous exercise during the 2 days preceding their admission to CRSU. Blood samples, including complete blood count, hepatic panel, lipid profile, haemoglobin A1c and immunosuppressant levels, were collected. After lunch, patients entered a whole room indirect calorimeter (respiration chamber) for a total of 18 h; the characteristics of the whole room indirect calorimeter used in this study are reported elsewhere.^18^ After 6 h, all patients were given a standardized meal (50% carbohydrates, 20% protein and 30% fat), and caloric requirements were individually calculated based on the patient’s height, weight and age using the Mifflin‐St. Jeor equation.^19^ Following the administration of the standardized meal, the patients continued to fast for an additional 12 h. Total energy expenditure (EE), CO_2_ production and O_2_ consumption were recorded every 1 min for a total of 18 h. While the patient was inside the chamber, only normal physical activity was allowed (i.e. no exercise). Mitochondrial fuel use was quantified by measuring whole‐body CO_2_ production relative to O_2_ consumption or respiratory quotient (RQ). The RQ oscillates between 0.7 and 1.0, which is indicative of either predominantly FA or glucose oxidation respectively.

Anthropometric measurements including height and weight were recorded at the time of admission to the CRSU. Body composition was quantified via magnetic resonance imaging (MRI). After an overnight fast, patients were scanned in a research‐dedicated Phillips Ingenia 3.0T MRI scanner using a 6‐min dual‐echo Dixon protocol, providing water‐ and fat‐separated volumetric data set covering neck to knees. Body composition profiling was performed using AMRA® Researcher.^20^ Acquired images were analysed for visceral adipose tissue (VAT), abdominal subcutaneous adipose tissue (ASAT), thigh fat‐tissue free muscle volume (FFMV) and muscle fat infiltration (MFI). The body compartments including VAT, ASAT and FFMV were standardized the height by dividing the body compartments by height squared (i.e. VATi, ASATi and FFMVi). For each subject, a personalized FFMVi *z*‐score (MVZ) was calculated. The MVZ measures how many standard deviations each subject deviate from the mean FFMVI of their matched control groups with the same gender and body size.^21^ To remove the known gender association of MFI, MFI was adjusted (MFI_adj_) by removing the gender‐specific mean MFI.^20^

### 2.3 Statistical analysis

Data are presented as means with standard deviation or frequency and percentage as appropriate. The RQ (CO_2_ production to O_2_ consumption ratio) was plotted against time at 1‐min intervals for 18 h. The RQ curves were smoothed using local linear regression and the smoothing parameter was selected via cross‐validation. This was subsequently modelled with RQ on the *y*‐axis and time on the *x*‐axis using a cubic B‐spline to better fit the data. The graph was interrogated to identify biologically relevant times points that included RQ at the time of standardized meal administration (180 min). To determine efficient and maximal carbohydrate utilization, the time to peak RQ after administration of standard meal was determined. Next, to determine if both groups had the equal capacity to utilize carbohydrates, the peak RQ between the two groups was compared. To determine the whole‐body FA oxidation during the fasting state, RQ 360 min after a standardized meal was compared between the two groups as well as the lowest RQ after meal administration. To better understand the relationship between biofuel utilization (i.e. RQ) and bioclinical parameters, linear model of residuals of the cubic B‐spline models against gender, diabetes, immunosuppression (tarcrolimus use), age, body mass index (BMI), FFMVi, VATi, and MFI_adj_ were generated.

To better understand the relationship between obesity, NAFLD and metabolic flexibility (i.e. fasting RQ), a staged analysis was completed. In the first step, the relationship between NASH diagnosis and BMI was evaluated by using both as co‐variates in predicting fasting RQ in the entire cohort. Next, to better understand the interplay between NASH diagnosis and BMI, subgroup analysis was performed in patients transplanted for NASH cirrhosis in which BMI as a predictor of fasting RQ was performed using multiple linear regression. In the final step, in subgroup analysis in a cohort of patients receiving LT for NASH cirrhosis, liver fat as measured by MRI‐PDFF and BMI were used as co‐variates in predicting fasting RQ using multiple linear regression.

For all patients, EE was measured directly at 1‐min intervals in the whole room calorimeter. EE was graphed versus time and smoothed using local linear regression and the smoothing parameter was selected via cross‐validation. Resting EE (REE) was measured in the fasting state, while the subjects were resting in a quiet surrounding at 24°C. The REE curves of the two groups were compared. The relationship between REE and gender, diabetes, immunosuppression (tarcrolimus use), age, BMI, FFMVi, VATi and MFI_adj_ was evaluated using linear models of the cubic B‐spline models. A nominal *p*‐value of <.5 was considered statistically significant.

## 3 RESULTS

### 3.1 Patient characteristics

The study cohort consisted of 45 subjects that underwent LT for NASH (*n* = 35) and non‐NASH (*n* = 10) indications. The mean age of patients transplanted for NASH vs. non‐NASH cirrhosis was similar (61 ± 9 vs. 60 ± 12 years, *p* = .75). The cohorts were similar with regard to gender and ethnicity (Table 1). While the prevalence of hypertension was similar across the two cohorts, patients transplanted for NASH cirrhosis were more likely to have diabetes and dyslipidemia (Table 1). Serum aminotransferase, alkaline phosphatase and bilirubin levels were similar between the two cohorts. Expectedly, patients transplanted for NASH cirrhosis had lower serum high density lipoprotein cholesterol (HDL‐C) (43 ± 12 vs. 53 ± 10 mg/dl, *p* = .02) and higher triglyceride (170 ± 112 vs. 102 ± 77, *p* = .043) levels. Finally, the two cohorts were similar with regard to transplant‐related metrics including time from LT and immunosuppressant use.

**TABLE 1:** Patient characteristics of the study cohort

	LT for NASH cirrhosis (*n* = 35)	LT for non‐NASH cirrhosis (*n*‐10)	*p* value
Demographics			
Age (years)	61 ± 9	60 ± 12.	.75
Gender (% female)	28	40	.47
Ethnicity (% Caucasian)	89	80	.49
Co‐morbidities			
Body mass index (kg/m^2^)	37.1 ± 5.5	26.2 ± 5.0	<.0001
Diabetes (%)	53	10	.03
Hypertension (%)	89	90	1.0
Dyslipidemia (%)	81	40	.02
Laboratory			
ALT (U/L)	34 ± 25	26 ± 15	.30
AST (U/L)	30 ± 14	28 ± 10	.59
Alkaline phosphatase (U/L)	102 ± 36	144 ± 64	.08
Bilirubin (mg/dl)	0.82 ± 0.40	0.59 ± 0.17	.09
Creatinine (mg/dl)	1.27 ± 0.41	1.31 ± 0.29	.75
Haemoglobin A1c (%)	6.0 ± 1.3	5.2 ± 0.5	.08
Lipid profile			
HDL‐C (mg/dl)	43 ± 12	53 ± 10	.02
LDL‐C (mg/dl)	84 ± 25	101 ± 26	.10
Total cholesterol (mg/dl)	159 ± 27	174 ± 36	.17
Triglycerides (mg/dl)	170 ± 112	102 ± 77	.043
Time from LT (months, IQR)	34 (21, 94)	52 (15, 86)	.31
Tacrolimus (%)	81	70	.57
Cyclosporine (%)	17	30	.57

Abbreviations: ALT, alanine aminotransferase; AST, aspartate aminotransferase; LDL‐C, low density lipoprotein cholesterol; LT, liver transplantation; NASH, non‐alcoholic steatohepatitis.

### 3.2 Body composition

**FIGURE 1:**
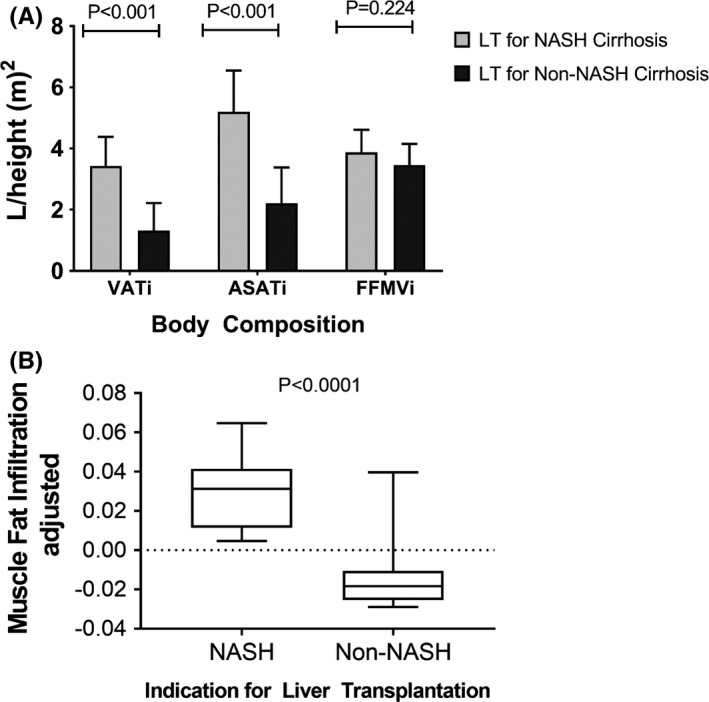
(A) Body compartment of the study cohort (ASATi, abdominal subcutaneous adipose tissue standardized to a height; FFMVi, fat‐free mass value standardized to a height; VATi, visceral adipose tissue standardized to height). (B) Patients transplanted for non‐alcoholic steatohepatitis (NASH) cirrhosis have greater muscle fat infiltration compared to patients transplanted for non‐NASH cirrhosis. LT, liver transplantation

Patients transplanted for NASH cirrhosis had a higher BMI compared to those transplanted for non‐NASH cirrhosis (37.1 ± 5.5 vs. 26.2 ± 5.0 kg/m^2^; *p* < .0001). Similarly, patients transplanted for NASH cirrhosis compared to non‐NASH cirrhosis had higher VATi, ASATi and lower FFMVi (Figure 1A). Patients receiving LT for NASH cirrhosis had significantly higher MFI than patients transplanted for non‐NASH cirrhosis (Figure 1B). In multivariate analysis, FFMVi was positively associated with BMI and tacrolimus (vs. cyclosporine use) and inversely with female gender and presence of diabetes (Table 2). MFI was positively associated with BMI and diabetes. The ASATi was positively associated with male gender, BMI and presence of diabetes but none of the other parameters including immunosuppression and time from LT. Finally, VATi was directly associated with BMI and cyclosporine use.

**TABLE 2:** The association between respiratory quotient and bioclinical parameters in the study cohort during post‐prandial and fasted state (BMI; body mass index, FFMVi; fat‐free mass index, MFI; muscle fat infiltration, VATi; visceral adipose tissue index)

	NASH			Non‐NASH			*p* value NASH versus non‐NASH
	Estimate	*SE*	*p* value	Estimate	*SE*	*p* value	
Fed state							
Gender (male)	.026	.003	<.001	.056	.001	<.001	<.001
Diabetes	−.068	.003	<.001	−.258	.007	<.001	<.001
Age (years)	.018	.001	<.001	.017	.001	<.001	.9045
BMI (kg/m^2^)	−.002	.002	.309	.049	.003	<.001	<.001
Tacrolimus	.053	.002	<.001	−.011	.001	<.001	<.001
FFMVi	−.047	.002	<.001	−.014	.002	<.001	<.001
MFI	.002	.001	.014	.075	.003	<.001	.4301
VATi	.022	.001	<.001	−.042	.002	<.001	<.001
Fasting state							
Gender (male)	.015	.002	<.001	.048	.001	<.001	<.001
Diabetes	−.043	.002	<.001	−.266	.006	<.001	<.001
Age (years)	.019	.001	<.001	.012	.001	<.001	<.001
BMI (kg/m^2^)	.017	.001	<.001	.011	.002	<.001	.2768
Tacrolimus	.028	.02	<.001	−.004	.001	<.001	<.001
FFMVi	−.034	.002	<.001	.029	.002	<.001	<.001
MFI	−.001	.0005	.006	.112	.003	<.001	<.001
VATi	.006	.001	<.001	−.038	.002	<.001	<.001

### 3.3 Metabolic flexibility

**FIGURE 2:**
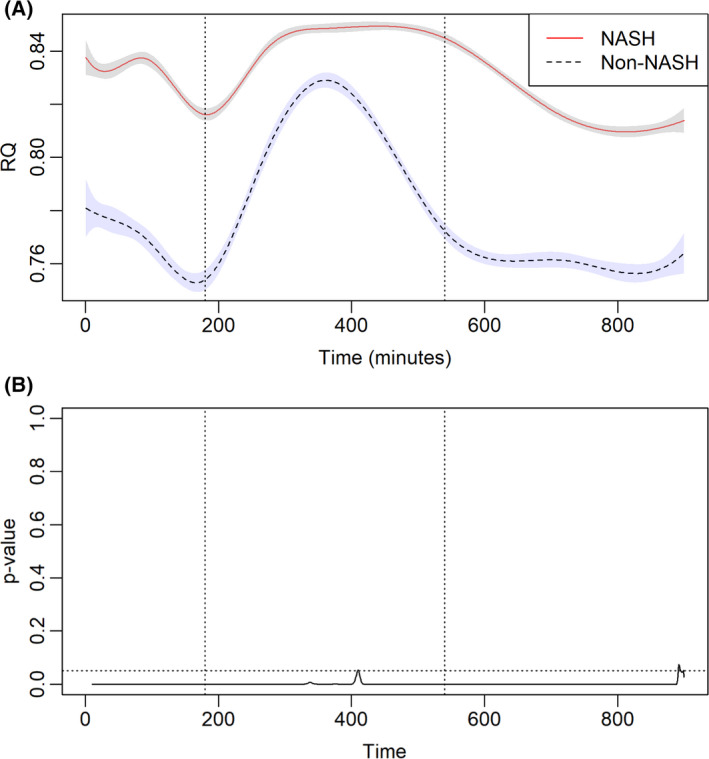
(A) Respiratory quotient (RQ) in patients receiving liver transplantation for non‐alcoholic steatohepatitis (NASH) and non‐NASH cirrhosis. The dotted line represents a standardized meal. (B) The corresponding *p* value for RQ is depicted in (A). Horizontal dotted line represents *p* value = .05. Vertical dotted lines represent a standardized meal and 360 min after that

The whole‐body energy utilization in patients transplanted for NASH vs. non‐NASH cirrhosis is depicted in Figure 2. Immediately prior to standardized meal administration, patients transplanted for NASH cirrhosis had higher baseline RQ (*p* < .05). Post‐prandially, the RQ increased for both cohorts, indicative of increasing carbohydrate utilization following a standardized meal. In the post‐prandial period, the time to reach RQ peak or maximal carbohydrate metabolism was similar in patients transplanted for NASH vs. non‐NASH cirrhosis (95% CI: 416 ± 238 vs. 385 ± 144 min, *p* = .39). However, patients in the NASH cohort had a higher peak RQ (95% CI: 0.849 ± 0.002 vs. 0.829 ± 0.003 min, *p* < .001), indicative of greater carbohydrate utilization. Furthermore, patients in the NASH cohort had higher remained at peak RQ significantly longer than the non‐NASH cohort (*p* < .001; Figure 2A,B).

As patients transitioned from post‐prandial state to fasting state, a decline in RQ indicative of biofuel switch to FA oxidation was noted in all patients (Figure 2A). With the transition to fasted state, the decline noted in RQ was rapid in the non‐NASH cohort but remained significantly elevated in the NASH cohort. In the fasting state, the RQ in patients transplanted for NASH cirrhosis was 0.845 (95% CI 0.843, 0.847) significantly higher compared to 0.772 (95% CI 0.769, 0.775) observed in patients transplanted for non‐NASH cirrhosis (*p* < .001). Furthermore, the lower RQ persisted in patients throughout the fasting state. This higher RQ observed in patients with NASH cirrhosis was indicative of greater reliance on carbohydrate metabolism in the fasted state (i.e. metabolic inflexibility) in patients transplanted for NASH cirrhosis.

### 3.4 Risk factors for reduced metabolic flexibility

To gain a deeper understanding of clinical predictors of metabolic flexibility, we generated a linear model in which RQ overtime was correlated with clinical and body composition parameters (Table 2). In the post‐prandial state, in patients transplanted for NASH cirrhosis, RQ correlated positively with male gender, age, tacrolimus use, VATi and MFI and negatively with the presence of diabetes and FFMVi. In patients receiving LT for non‐NASH indications, post‐prandial RQ correlated positively with male gender, age and MFI but negatively with the presence of diabetes, tacrolimus use (vs cyclosporine), FFMVi and VATi. In the fasted state, in patients receiving LT for NASH cirrhosis, RQ correlated positively with male gender, age and VATi. In patients receiving LT for non‐NASH indications, in the fasted state RQ positively correlated with male gender, age, FFMVi and MFI.

### 3.5 Relationship between BMI, liver fat and metabolic flexibility

**FIGURE 3:**
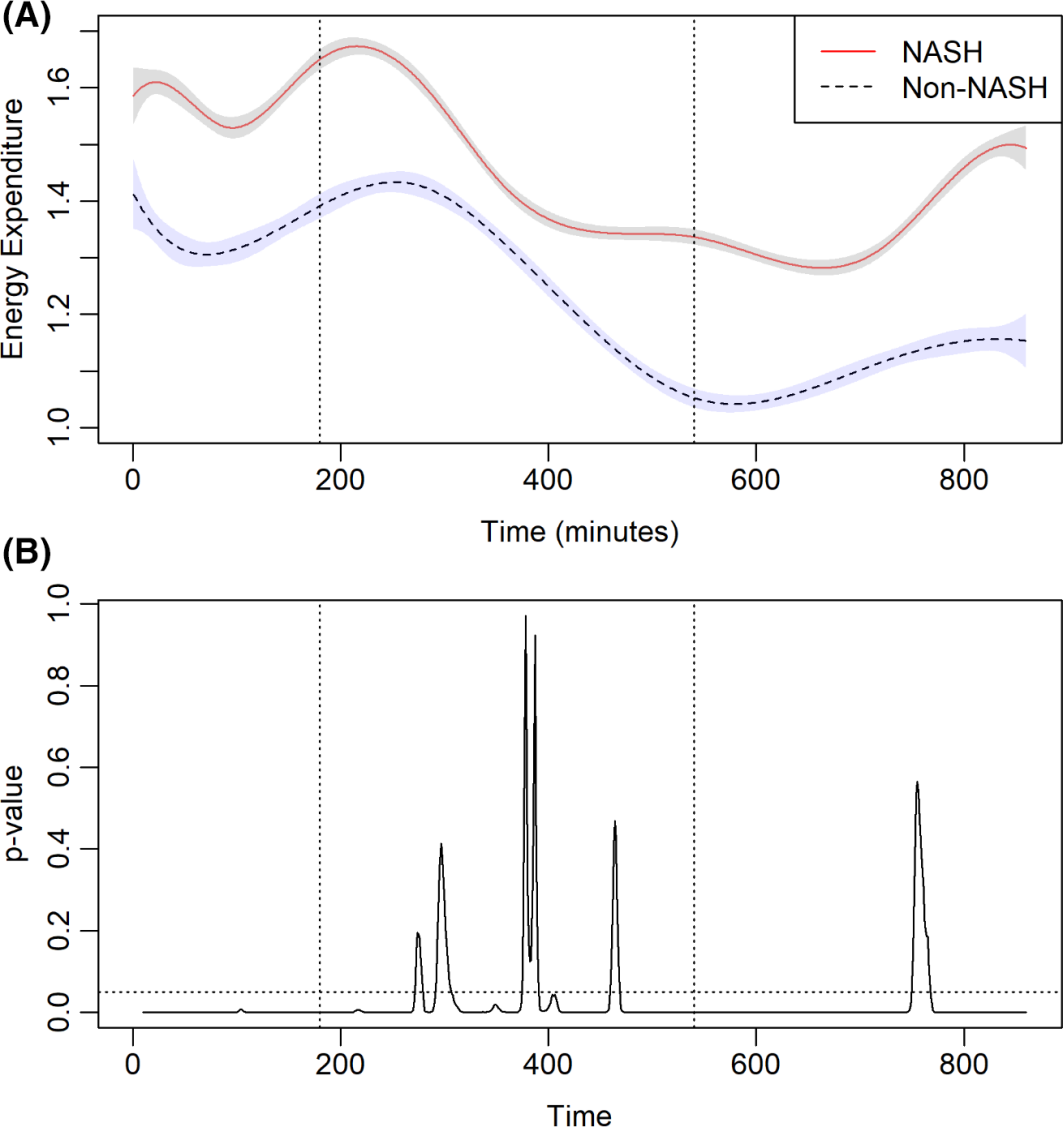
(A) Metabolic rate (MR) in patients receiving liver transplantation for non‐alcoholic steatohepatitis (NASH) and non‐NASH cirrhosis. The dotted line represents a standardized meal. (B) The corresponding *p* value for MR is depicted in Figure 2a. The dotted line represents *p* value = .05. Vertical dotted lines represent standardized meal and 360 min after that

In this analysis, using the whole cohort, BMI and NASH diagnosis were used as predictors of fasting RQ in the entire cohort. NASH as an aetiology of cirrhosis contributed to the fasting RQ (standardized *β*‐coefficient: 0.0734 ± 0.0190, *p* = .0004) but not BMI (standardized *β*‐coefficient: −0.0017 ± 0.0079, *p* = .8), indicating NASH diagnosis is independently associated with metabolic flexibility but not BMI in the entire cohort. Next, in a subgroup analysis of patients transplanted for NASH cirrhosis, the relationship between BMI and fasting RQ was evaluated using regression analysis. BMI strongly correlated with fasting RQ (standardized *β*‐coefficient: 0.0267 ± 0.0090, *p* = .007), whereas liver fat content, as measured on MRI‐PDFF, did not (standardized *β*‐coefficient: −0.0064 ± 0.0090, *p* = .48), indicating that among patients transplanted for NASH cirrhosis, BMI is the key driver of reduced metabolic flexibility (Figure 3A,B).

### 3.6 Energy expenditure

**FIGURE 4:**
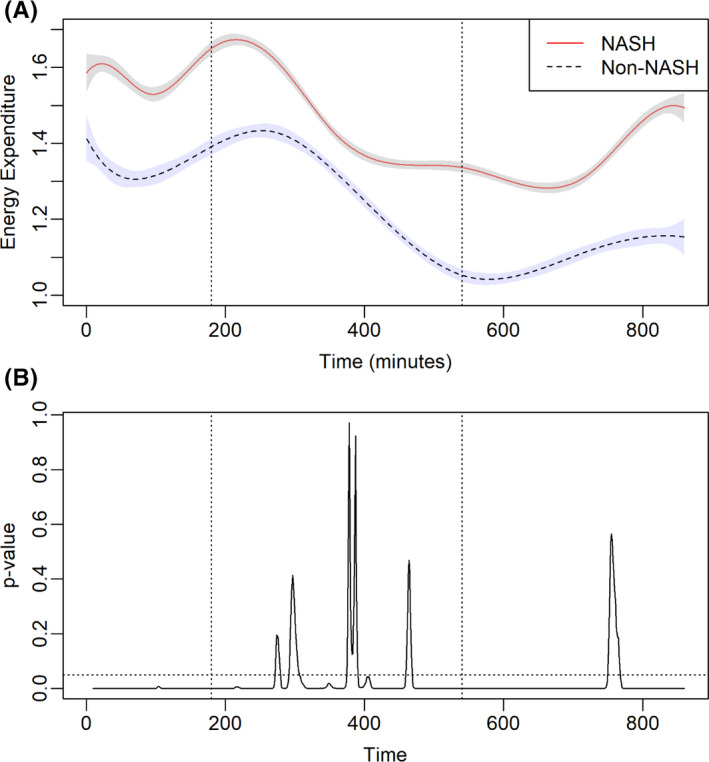
(A) Relationship between respiratory quotient (RQ) and body mass index in a subset of patients receiving liver transplantation (LT) for non‐alcoholic steatohepatitis‐related cirrhosis. (B) Relationship between RQ and liver fat content as measured by MRI‐PDFF in a subset of patients receiving LT for non‐alcoholic steatohepatitis‐related cirrhosis

Energy expenditure was measured directly for each participant within the whole room calorimeter (Figure 4). At entry into the whole room calorimeter, EE was higher for patients transplanted for NASH vs. non‐NASH cirrhosis. In the fed state, EE was similar between the two groups. With fasting, the EE continued to decline in both cohorts, however, patients transplanted for NASH cirrhosis had higher REE. In patients transplanted for NASH cirrhosis, a positive relationship between REE and male gender, presence of diabetes, BMI, FFMVi and VATi was demonstrated (Table 3). In patients transplanted for non‐NASH indications, REE positively correlated with male gender, FFMVi and VATi.

**TABLE 3:** Association between resting energy expenditure and bioclinical parameters (BMI, body mass index; FFMVi, fat‐free mass index; MFI, muscle fat infiltration; VATi, visceral adipose tissue index)

	NASH			Non‐NASH			*p* value NASH versus non‐NASH
	Estimate	*SE*	*p* value	Estimate	*SE*	*p* value	
Gender (male)	.354	.027	<.001	.119	.006	<.001	<.001
Diabetes	.393	.031	<.001	−.023	.032	.459	<.001
Age (years)	−.263	.013	<.001	−.064	.003	<.001	<.001
BMI (kg/m^2^)	.039	.019	.038	−.173	.011	<.001	<.001
Tacrolimus	−.550	.022	<.001	−.100	.006	<.001	<.001
FFMVi	.147	.023	<.001	.121	.008	<.001	<.001
MFI	−.034	.007	.006	−.008	.013	.515	<.001
VATi	.064	.008	<.001	.184	.009	<.001	<.001

## 4 DISCUSSION

In the present study, we demonstrate that patients transplanted for NASH cirrhosis have reduced metabolic flexibility, which correlates with the quality of skeletal muscle. Physiologically, the body readily transitions between carbohydrate metabolism during the fed state to FAs during the fasted state and reflects the relative abundance of these biofuels during these states.^11^ Rapid transitions between available substrates are possible because of the ability of mitochondria to readily utilize the most abundantly available biofuel.^9^ Thus, the inability to do so reflects reduced skeletal muscle mitochondrial plasticity and is associated with metabolic co‐morbid conditions such as obesity and diabetes. This reduced mitochondrial flexibility was apparent initially during the post‐prandial phase when patients transplanted for NASH cirrhosis had a slower and more gradual increase in RQ demonstrating a sluggish transition to carbohydrate metabolism after meal ingestion. Similarly, as patients transitioned to the fasted state, patients transplanted for non‐NASH cirrhosis had a rapid decline in RQ, whereas in patients transplanted for NASH cirrhosis, the decline in RQ was more gradual. In pre‐clinical studies, sluggish mitochondrial response to substrate selection is observed at the level of gene and protein expression and robust changes in a wide range of transcripts that occur during fed the state is attenuated.^22^ These responses likely reflect sub‐optimal cellular anticipation of priming the mitochondrial machinery for the next meal. Additionally, the differences in whole‐body utilization are also likely reflective of different metabolic phenotype that is present in patients at the time of LT and thus predisposing patients transplanted for NASH to developing recurrence of NAFLD post‐LT. These findings potentially underscore the importance of non‐hepatic factors as drivers of recurrence of post‐LT NAFLD, which may potentially be an innocent bystander and perturbed metabolic milieu of patients transplanted for NASH cirrhosis.

### 4.1 Relationship between weight, NALFD and metabolic flexibility

The clinical impact of reduced metabolic flexibility is higher recycling of FA, which can potentially lead to adiposity and obesity when deposited in adipose tissues, NAFLD recurrence when it occurs in the liver and MFI when it occurs in skeletal muscle.^4^, ^20^ The exact mechanism underlying reduced metabolic flexibility is not known, however, it likely results from metabolic co‐morbidities, which are exacerbated further by exposure to chronic immunosuppression. Using imaging‐based quantification of liver fat content (i.e. MRI‐PDFF), we demonstrated that reduced metabolic flexibility is present even before patients develop post‐LT NAFLD and the severity of NAFLD does not influence the severity of metabolic *(in)*flexibility. Rather, it is weight and adiposity that are key predictors of metabolic flexibility in patients transplanted for NASH cirrhosis. These data suggest that the development of metabolic flexibility might precede the development of NAFLD following LT and therefore has the potential to serve as a novel therapeutic target for the treatment of post‐LT NAFLD. However, additional studies with longitudinal and translational study designs are necessary to better understand the relationship between metabolic flexibility and the development of NAFLD following LT.

### 4.2 REE on transplant recipients

REE is the largest contributor to daily EE and is defined as energy expended at thermoneutrality in the fasting state when not performing physical work. In the present study, we demonstrate that patients transplanted for NASH cirrhosis have higher REE compared to patients transplanted for non‐NASH. While this may seem contrary to popular belief, these findings are in accordance with published literature demonstrating that obesity is associated with higher REE because of elevated increased fat‐free mass and body fat that is common in patients with obesity.^23^ These findings were demonstrated in body composition analysis, which demonstrated higher fat and fat‐free mass in subjects transplanted for NASH cirrhosis. Furthermore, it is possible that in patients transplanted for NASH cirrhosis have a higher contribution from energy‐requiring metabolic pathways, such as gluconeogenesis, de novo lipogenesis, triglyceride synthesis which require significantly higher energy to maintain and have been implicated in the pathogenesis of NAFLD.^24^ Interestingly, no difference in EE was observed between the two groups in the standardized meal, suggesting that the NASH group has an impaired thermic effect of food. This could lead to decreased dissipation of energy further promoting weight gain and insulin resistance, facilitating the recurrence of NASH.^25^

### 4.3 Strengths and limitations

The findings from our observations must be evaluated in the context of their limitations. By design, this is a proof of concept study to evaluate biofuel utilization after LT. Thus, these findings should not be extrapolated to non‐LT patients with NASH. Furthermore, patients were dichotomized into NASH vs. non‐NASH, however, additional prospective studies are required to provide granularity on how other causes of chronic liver disease (i.e. alcohol, hepatitis C, etc.) may impact metabolic flexibility post‐LT. The current study enrolled patients on calcineurin inhibitors, which is reflective of the transplant population at large, but it was not designed to evaluate the impact of a specific type of immunosuppression as the metabolic profile of various immunosuppressants can be significantly different. Owing to the cross‐sectional nature of the current study, it is unclear how metabolic inflexibility might impact clinical outcomes such as cardiovascular risk, development of post‐LT NAFLD and survival. These clinically significant endpoints and metabolic flexibility need to be better defined but require adequately powered prospective studies.

In conclusion, we demonstrated that patients transplanted for NASH cirrhosis have impaired biofuel utilization which is associated with skeletal muscle structure. These findings underscore the importance of better defining the pathophysiology of post‐LT weight gain and obesity so as to provide targeted therapy with the long‐term goal of improving outcomes in LT recipients.

## DISCLOSURES

None: SP, ATB, SS, SB, SC, CSB, FSC; MSS: Bristol Myers Squib (consulting); AMRA (Advising); AJS: None for this project. Dr Sanyal is President of Sanyal Biotechnology and has stock options in Genfit, Akarna, Tiziana, Indalo, Durect Inversago and Galmed. He has served as a consultant to AstraZeneca, Nitto Denko, Conatus, Nimbus, Salix, Tobira, Takeda, Jannsen, Gilead, Terns, Birdrock, Merck, Valeant, Boehringer‐Ingelheim, Bristol Myers Squibb, Lilly, Hemoshear, Zafgen, Novartis, Novo Nordisk, Pfizer, Exhalenz and Genfit. He has been an unpaid consultant to Intercept, Echosens, Immuron, Galectin, Fractyl, Syntlogic, Affimune, Chemomab, Zydus, Nordic Bioscience, Albireo, Prosciento and Surrozen. His institution has received grant support from Gilead, Salix, Tobira, Bristol Myers, Shire, Intercept, Merck, AstraZeneca, Malinckrodt, Cumberland and Novartis. He receives royalties from Elsevier and UptoDate.

## PATIENT CONSENT STATEMENT

All study participants gave written informed consent to participate in this study and have their results published as part of this study.

## Supporting information


Supinfo

Figure S1
