# Supplementary material for: Differential fuel utilization in liver transplant recipients and its relationship with non‐alcoholic fatty liver disease
Source: Liver Int. 2022 Feb 24;42(6):1401–9. doi: 10.1111/liv.15178 (PMC9189602; doi:10.1111/liv.15178)
Supplement: Supplementary file 1 — Supinfo [file LIV-42-1401-s002.docx]

**SUPPLEMENTARY MATERIAL**:

**Immunosuppression protocol**:

1. **Preserved Renal Function**
2. Corticosteroids are used in the peri-transplant period and discontinued as soon as calcineurin inhibitors (CNI) are initiated. This usually occurs during their initial hospitalization
3. Tacrolimus is the preferred CNI with the following trough targets:
   1. 0-3 months: 6-8 ng/mL
   2. 4-12 months: 4-6 ng/mL
   3. >12 months: 2-4 ng/mL
4. Mycophenolate is started at the time of transplant with the following post-LT dosing:
   1. 0-6 months: 500mg bid
   2. 6-12 months: 250mg bid
   3. >12 months: consider discontinuation if no history of rejection and stable graft function.
5. **Renal Insufficiency**
6. Steroids: initiated on methylpredinsone, which is converted to prednisone and tapered off by 4 weeks post-LT
7. Tacrolimus: started within 1 week of LT with the following trough targets:
   1. 0-12 months: 4-6 ng/mL
   2. >12 months: 2-4 ng/mL
8. Mycophenolate is started at the time of LT with the following post-LT dosing:
   1. 0-3 months: 1000mg bid
   2. 3-6 months: 500mg bid
   3. 6-12 months: 250mg bid
   4. >12 months: consider discontinuation if no history of rejection and stable graft function.
