# Supplementary figures and images for: Differential fuel utilization in liver transplant recipients and its relationship with non‐alcoholic fatty liver disease
Source: Liver Int. 2022 Feb 24;42(6):1401–9. doi: 10.1111/liv.15178 (PMC9189602; doi:10.1111/liv.15178)

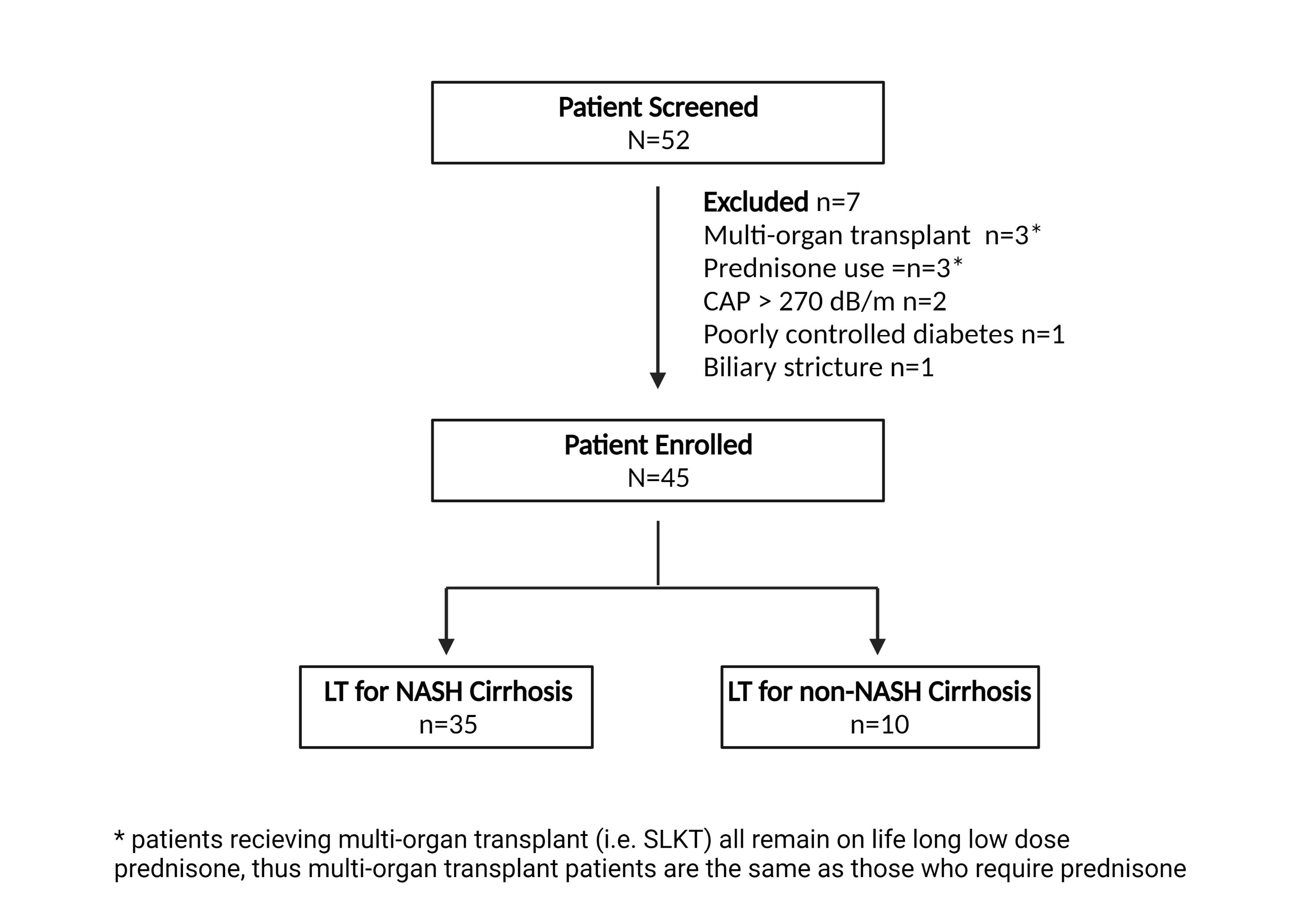

Supplement: Supplementary file 2 — Figure S1 [file LIV-42-1401-s001.tiff]
