# Supplementary material for: Differential fuel utilization in liver transplant recipients and its relationship with non-alcoholic fatty liver disease
Source: Liver Int. Author manuscript; Available in PMC 2023 Jun 1. (PMC9189602; doi:10.1111/liv.15178)
Supplement: Supplementary file 1 [file NIHMS1794021-supplement-Supplemental_Information.docx]

**SUPPLEMENTARY MATERIAL**:

**Immunosuppression protocol**: 

I. **Preserved Renal Function** 

A. Corticosteroids are used in the peri-transplant period and discontinued as soon as calcineurin inhibitors (CNI) are initiated. This usually occurs during their initial hospitalization

B. Tacrolimus is the preferred CNI with the following trough targets: 

a. 0-3 months: 6-8 ng/mL 

b. 4-12 months: 4-6 ng/mL 

c. >12 months: 2-4 ng/mL 

C. Mycophenolate is started at the time of transplant with the following post-LT dosing:

a.  0-6 months: 500mg bid 

b. 6-12 months: 250mg bid 

c. >12 months: consider discontinuation if no history of rejection and stable graft function. 

II. **Renal Insufficiency** 

A. Steroids: initiated on methylpredinsone, which is converted to prednisone and tapered off by 4 weeks post-LT

B. Tacrolimus: started within 1 week of LT with the following trough targets:

a. 0-12 months: 4-6 ng/mL

b. >12 months: 2-4 ng/mL

C. Mycophenolate is started at the time of LT with the following post-LT dosing:

a. 0-3 months: 1000mg bid 

b. 3-6 months: 500mg bid 

c. 6-12 months: 250mg bid 

d. >12 months: consider discontinuation if no history of rejection and stable graft function.
